# Biocompatibility of Poly(acrylonitrile-butadiene-styrene) Nanocomposites Modified with Silver Nanoparticles

**DOI:** 10.3390/polym10111257

**Published:** 2018-11-13

**Authors:** Magdalena Ziąbka, Michał Dziadek, Elżbieta Menaszek

**Affiliations:** 1 AGH University of Science and Technology, Faculty of Materials Science and Ceramics, Department of Ceramics and Refractories, al. Mickiewicza 30, 30-059 Krakow, Poland; 2 AGH University of Science and Technology, Faculty of Materials Science and Ceramics, Department of Glass Technology and Amorphous Coatings, al. Mickiewicza 30, 30-059 Krakow, Poland; dziadek@agh.edu.pl; 3 Jagiellonian University, Collegium Medicum, Faculty of Pharmacy, Department of Cytobiology, ul. Medyczna 9, 30-688 Krakow, Poland; elzbieta.menaszek@uj.edu.pl

**Keywords:** nanocomposites, biocompatibility, cell viability, fibroblasts, osteoblasts

## Abstract

We evaluated the biological, mechanical, and surface properties of polymer nanocomposites manufactured via plastics processing, extrusion, and injection moulding. The aim of this study was to identify the interaction of fibroblasts and osteoblasts with materials intended for middle ear implants. We examined if silver nanoparticles (AgNPs) may change the mechanical parameters of the polymer nanocomposites. In our study, the biostable polymer of thermoplastic acrylonitrile-butadiene-styrene (ABS) copolymer was used. Silver nanoparticles were applied as a modifier. We discuss surface parameters of the materials, including wettability and roughness, and evaluated the microstructure. The mechanical parameters, such as the Young’s modulus and tensile strength, were measured. Cytotoxicity tests were conducted on two cell lines: Hs680.Tr human fibroblasts and Saos-2 human osteoblasts. Cell viability, proliferation, and morphology in direct contact with nanocomposites were tested. Based on the results, the incorporated modifier was found to affect neither the number of osteoblasts nor the fibroblast cells. However, the addition of AgNPs had a relatively small effect on the cytotoxicity of the materials. A slight increase in the cytotoxicity of the test materials was observed with respect to the control, with the cytotoxicity of the materials tending to decrease after seven days for osteoblast cells, whereas it remained steady for fibroblasts. Based on optical microscope observation, the shape and morphology of the adhered cells were evaluated. After seven days of culture, fibroblasts and osteoblasts were properly shaped and evenly settled on the surface of both the pure polymer and the silver nanoparticle-modified composite. Water droplet tests demonstrated increased hydrophilicity when adding the AgNPs to ABS matrices, whereas roughness tests did not show changes in the surface topography of the investigated samples. The 0.5% by weight incorporation of AgNPs into ABS matrices did not influence the mechanical properties.

## 1. Introduction

Nanoparticle-polymer composites have potential for application as the next generation of instructive biomaterials. They may display parameters comparable to casual composites, potentially allowing the development of modern devices for antibacterial treatment, medical imaging, tissue engineering, drug delivery, cancer therapy, and dental applications [1,2,3]. Obtaining optimal biological, mechanical, physical, and chemical properties is critical during the development of materials, especially for materials that will be implanted for more than 30 days. Medical devices, including implants, are classified with regard to the level of risk posed to the human body due to their implantation according to the definitions and rules of the 93/42/EEC Directive of the European Parliament and of the Council, also known as the Medical Devices Directive (MDD) [4]. This classification is simple: the higher the class, the higher the risk for the person after implantation. Classification may vary depending on the usage or dosage. All products that are supposed to be implanted for less than 30 days are classified in the second group. Implantation for more than 30 days classifies them into the third class. Additionally, even if a product will be implanted for a short period of less than 30 days but contains a drug that may affect healing results, it is assigned to the third class. Consequently, all materials developed to be integrated in the human body should be safe and biocompatible. Measurements of biological reactions and parameters assessing the possibility of using certain materials have been gathered in International Standard ISO 10993 [5]. Considering all of the above, during the development of a material, all properties cannot be worse at the end of an experiment than at the beginning—they should improve from the starting point. The addition of even a small amount of nanoparticles (NPs) to the polymer matrix and then adding the nanocomposite to the body will affect the interaction of nanoparticles with cells. Interaction with immune cells may result in molecular reactions, which may lead to higher sensitivity to disease and cancer growth [6]. Use of nanoparticles may not only affect various reactions within cells but also cause undesired allergic reactions [7,8,9]. Modification of composites with silver (Ag) nanoparticles may influence their antibacterial properties. Silver nanoparticles (AgNPs) are biocidal and their mechanism of toxicity is related to the damage of cell membranes and oxidative stress [10,11]. Silver ions can cause deformation of the protein structure by binding to them via disulphide bonds in the cytoplasm. Since the malformed proteins are incorporated into the plasma membrane, cell permeability is altered, leading to cell death [12]. Understanding how a nanocomposite reacts with cells is a challenge from a toxicological point of view, but is crucial for future solutions in biomedicine. Cell research including human cell lines may provide information on the biocompatibility of nanomaterials [13].

The ideal middle ear prosthesis for ossicles reconstruction should be biocompatible, readily available, technically easy to use, and have the best possible biomechanical properties [14]. Considering biological factors, the prosthesis material ought to be antibacterial and stable in the environment, especially in the case of chronic middle ear inflammation. As far as the mechanical properties are concerned, the middle ear implant should retain its shape and measurements for a prolonged period of time, along with tensile strength and rigidity. The materials used for middle ear prostheses should be resistant to live load and fatigue conditions. Additionally, middle ear implants have to be adaptable to the micro-movements between the tympanic membrane and the middle ear [15]. Many different materials have been used for ossiculoplasty. One of them is titanium due to its high biocompatibility, low weight, and ease of use [16,17]. Polymeric prostheses made of polytetrafluoroethylene (PTFE) [18] and ceramic prostheses made of aluminum oxide ceramic (Al_2_O_3_) [19] or bioactive hydroxyapatite (HA) [20] are also used. Composite prostheses, such as Plastipore, PTFE, Polycel (thermal-fused Plastipore), HAPEX (polyethylene composites reinforced with HA), Flex-HA (a mixture of silastic—silicone plastic—and HA) and those created by Bojrab et al. [21] and Hahn et al. [22] are available in the medical devices market. However, none of these currently available alloplastic materials fulfill the antibacterial requirement.

The use of autogenic ossicles is not recommended in the case of chronic middle ear infection, otosclerosis, or cholesteatoma, since they contribute to the recurrence of the underlying disease [23]. As such, alloplastic materials, especially with bactericidal properties, seem to be a promising alternative to the aforementioned illnesses. Our previous results [24] showed that a middle ear prosthesis made of an ABS matrix and silver nanoparticles had antibacterial activity against Gram-positive and Gram-negative bacteria and was biocompatible in long-lasting tests on animals. Additionally, we proved that AgNPs accelerate the healing process of surrounded tissues, which is crucial to the length of convalescence after the reconstruction of ossicle chain.

In this manuscript, we present how AgNPs affect the physicochemical and biological properties of polymer composites. Given that the scientific literature describes the toxic action of silver nanoparticles, we wanted to determine if the proposed amount of the additive is safe for materials designed for otolaryngology.

## 2. Materials and Methods

### 2.1. Material Manufacturing

Two commercially-available polymers—ABS poly(acrylonitrile-butadiene-styrene)-Novodur HD 15 (INEOS Styrolution, Frankfurt, Germany; ABS-N) and Elix M205FC (Elix Polymers, La Canonja, Spain; ABS-E)—as well as composite materials modified with 0.5 wt % AgNPs (NanoAmor, Katy, TX, USA) with a purity of 99.9%, 80-nm particle size, and density of 10.49 g cm^−3^ were shaped into 10-mm-diameter discs. Polymer and composite materials were manufactured using plastics processing methods, extrusion, and injection moulding. The procedure for obtaining specimens was as follows. First, the granulates were prepared and dried in the laboratory dryer at 80 °C for 6 h. Next, the AgNPs were incorporated and homogenized with polymer granules in the plasticizing chamber using a 0.8-m-long screw at a homogenization temperature of 240 °C. Subsequently, the material was injected into the steel moulding form, cooled, and extracted. The injection parameters were selected and adapted for the process according to data sheet of the polymer manufacture. Injection temperature in the three zones was 240 °C, injection pressure was 80 kg cm^−2^, and flow was 80%.

### 2.2. Material Evaluation

#### 2.2.1. Scanning Electron Microscopy

A Nova NanoSEM 200 scanning electron microscope (SEM; FEI, Eindhoven, The Netherlands) coupled with a Genesis XM X-ray microanalysis system (EDAX, Tilburg, The Netherlands) featuring the Sapphire Si(Li) energy dispersive X-ray (EDX) detector was used to perform the detailed examination of the microstructure of the produced materials. The measurements and observations were conducted in high vacuum conditions, with back scatter electron detector (BSE) at an accelerated voltage of 10–18 kV. The samples were coated with a carbon layer.

#### 2.2.2. Atomic Force Microscopy

The topographical evaluation of the ABS-E, ABS-N, and their composites with AgNPs was performed via atomic force microscope (AFM, MultiMode 8 Bruker microscope, Karlsruhe, Germany), using antimony-doped silicon tips (spring constant = 40 N m^−1^), operating in tapping mode. Image analysis was performed using XEI 1.7.1 software (Park Systems, Suwon, Korea). Root mean square roughness (Rq) and the arithmetical mean roughness (Ra) values were calculated based on three AFM height images collected at three different places on the materials and are expressed as the mean ± standard deviation (SD).

#### 2.2.3. Surface Wettability

The surface wettability was evaluated by static water contact angle measurements. The contact angle was determined by the sessile drop method with an automatic drop shape analysis (DSA) system, DSA 10 Mk2 (Kruss GmbH, Hamburg, Germany). Ultrahigh quality (UHQ) water droplets of 0.25 μL were applied on each pure and dry sample. The experiments were carried out in constant temperature and humidity conditions. The apparent contact angle was calculated as an average of 10 measurements and is expressed as a mean ± standard deviation (SD).

#### 2.2.4. Tensile Test

Tensile strength (σ_M_) and Young’s modulus (E_t_) were determined using a universal testing machine, the Inspect Table Blue 5 kN with a 5-kN load cell (Hegewald&Peschke, Nossen, Germany). The pre-load force was 1 N, the test speed was 50 mm min^−1^. The samples for measurements were prepared according to EN ISO 527-1 [25]. Mechanical parameters were calculated by averaging 10 measurements and are expressed as mean ± SD.

#### 2.2.5. In Vitro Tests

In vitro biological evaluation of the produced materials was carried out using two cell lines: Hs680.Tr (human tracheal fibroblast; ATCC, Manassas, VA, USA) and Saos-2 (human osteosarcoma; ATCC, Manassas, VA, USA) from 3 passages. In order to obtain the cell suspension, the culture was washed twice with phosphate buffered saline (PBS), followed by a 5% trypsin solution with EDTA (HyClone, San Angelo, TX, USA). After washing and centrifugation, the cells were suspended in fresh medium. Next, 1 mL of the resulting cell suspension at a density of 10^4^ cells/mL was added to the wells of 48-well culture plates (Nunc, Roskilde, Denmark) containing sterile discs of the materials. The bottom surfaces of tissue culture polystyrene (TCPS) wells served as a control. TCPS also served as a negative control for cytotoxicity assays. Cell culture of the discs in direct contact with the test materials was carried out for 3 and 7 days of culture.

ToxiLight 100% Lysis Reagent set (Lonza, Walkersville, MD, USA) was used to disintegrate the cytomembrane of the cultured cells. Then a ToxiLight Bioassay Kit (Lonza, Walkersville, MD, USA) was used in order to establish the entire number of cells confirming their viability and proliferation. The amount of the released adenylate kinase was assessed with a PolarStar Omega reader (BMG Labtech, Ortenberg, Germany). Furthermore, the metabolic activity of the cells after 3 and 7 days of culture was evaluated using the PrestoBlue^®^ Cell Viability Reagent (Invitrogen, Waltham, MA, USA). PrestoBlue^®^ reagent was added to each well with cells spread on TCPS and then materials were incubated for 2 h at 37 °C in a 5% CO_2_ moistened atmosphere. Fluorescence was measured at 560/590 nm (excitation/emission, respectively) using a POLARstar Omega microplate reader (BMG Labtech, Ortenberg, Germany). Results are expressed as the mean ± SD from 8 measurements for each group.

Cytotoxicity of the materials was assessed via the bioluminescence method, using ToxiLight Bioassay Kit (Lonza, Walkersville, MD, USA), which measures the release of the enzyme adenylate kinase (AK) from damaged cells. AK is a robust protein present in all eukaryotic cells that is released into the culture medium when cells die. The luminescence was measured with a PolarStar Omega plate reader spectrophotometer (BMG Labtech, Ortenberg, Germany). The test was conducted on the supernatant from the cell culture and the results were compared to the entire enzyme concentration (proportional to the entire number of cells) released from all cells. Results are expressed as the mean ± SD from 8 measurements for each group.

Two samples of each series were used for morphological observation under a fluorescence optical microscope. The cells adhering to the materials were dyed for 1 min with acridine orange (AO) solution (Sigma, Saint Louis, MO, USA). After washing with PBS (HyClone, San Angelo, TX, USA), the cells were examined using an Olympus CX-41 (Olympus, Tokyo, Japan) with a fluorescence device. A digital E-520 camera (Olympus, Tokyo, Japan) was used to take photographs of the cells.

A Nova NanoSEM 200 scanning electron microscope (SEM; FEI, Eindhoven, The Netherlands) was used to perform a detailed morphological examination of the cells adhered to the investigated materials. The observations were conducted in high vacuum conditions, with a back scatter electron detector (BSE) at an accelerated voltage of 10–18 kV. After 7 days of cell culture, materials were rinsed with PBS and then cells were fixed with 3% glutaraldehyde solution in sodium cacodylate buffer at pH 7.4 (POCh, Gliwice, Poland) for 0.5 h. Subsequently, the cells were dehydrated in a graded series of ethanol solution (70%, 80%, 90%, 96%, and 100%) and dried in air. Cell morphologies were evaluated after coating with carbon.

#### 2.2.6. Statistical analysis

The results were analysed using one-way analysis of variance (ANOVA) with Duncan post hoc tests, which were performed with Statistica 10 (StatSoft^®^, Tulsa, OK, USA) software. The results were considered statistically significant when *p* < 0.05.

## 3. Results

### 3.1. SEM and AFM Measurements

**Figure 1:**
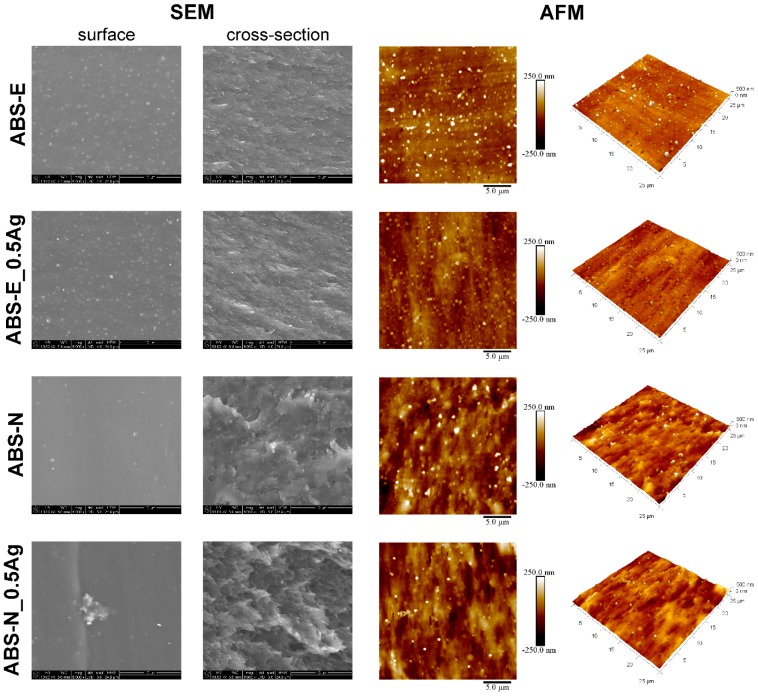
SEM and AFM tapping mode images of pure polymers and polymers containing silver nanoparticles.

AFM and SEM images (Figure 1) show the relevant differences in the surface morphology of the investigated materials. The both surfaces of ABS-E and ABS-N polymers and composite materials were rather homogenous. However, in the case of ABS-N materials, the surface appeared smoother in comparison to ABS-E materials. The SEM images of the pure ABS-E and its nanocomposite showed a grain-like structure, which was more visible in the AFM images. 

The ABS-N exhibited a smooth surface, whereas its cross-section was rougher compared to the ABS-N cross-section. We also observed small regions with silver aggregates. The aggregates were up to 5 micrometres in size.

**Figure 2:**
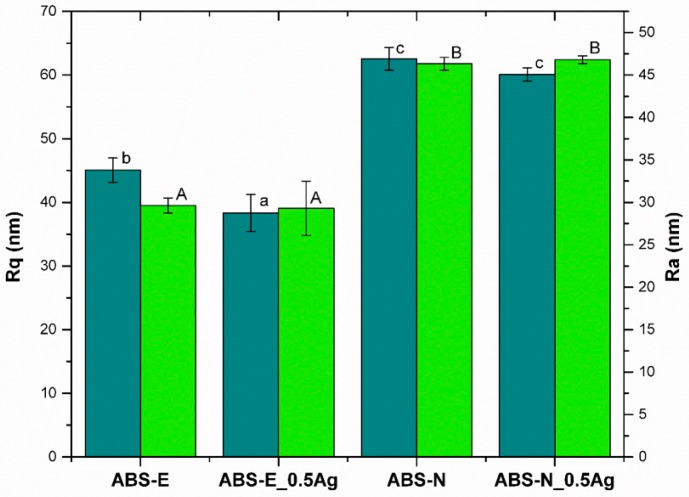
The root mean-square roughness (Rq) and average roughness (Ra) of pure polymers and polymers containing silver nanoparticles collected from AFM images. Statistically significant differences (*p* < 0.05) between the tested materials are marked a-c for Rq and A-B for Ra, respectively.

The arithmetic average values of the roughness profile (Ra) and root mean-square roughness (Rq), collected from AFM images, did not show significant changes upon modification of the polymer matrices with silver nano-additive (Figure 2). However, we observed slight differences in Rq measured for the ABS-E pure polymer and its composite, which might be related to the grain structure of the pure polymer, whereas the addition of AgNPs could influence this difference. The AFM measurements revealed that tested surfaces were characterized by Ra and Rq parameters below 65 nm in the case of ABS-N pure polymer and its composites, and below 45 nm for ABS-E materials. These results indicate the low surface roughness of the tested materials. 

### 3.2. Surface Wettability

**Figure 3:**
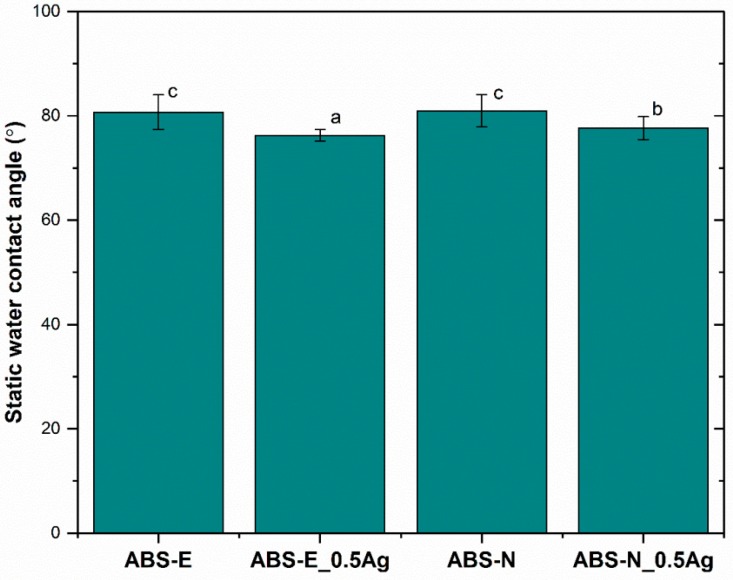
The static water contact angle of pure polymers and polymers containing silver nanoparticles. Statistically significant differences (*p* < 0.05) between the tested materials are marked a–c.

The contact angle measurements allowed us to evaluate the surface wettability of the tested samples. The results showed that the surfaces of all polymers and composite materials were hydrophilic. For both pure polymers (ABS-E and ABS-N), the wettability angle was estimated at the same level, below 85° (Figure 3). 

The incorporation of silver nanoparticles in the amount of 0.5 by weight into the polymer matrix slightly influenced the wettability of the composites. In comparison to polymers, composite materials were more hydrophilic and the wettability angle was below 80°.

### 3.3. Mechanical Properties

**Figure 4:**
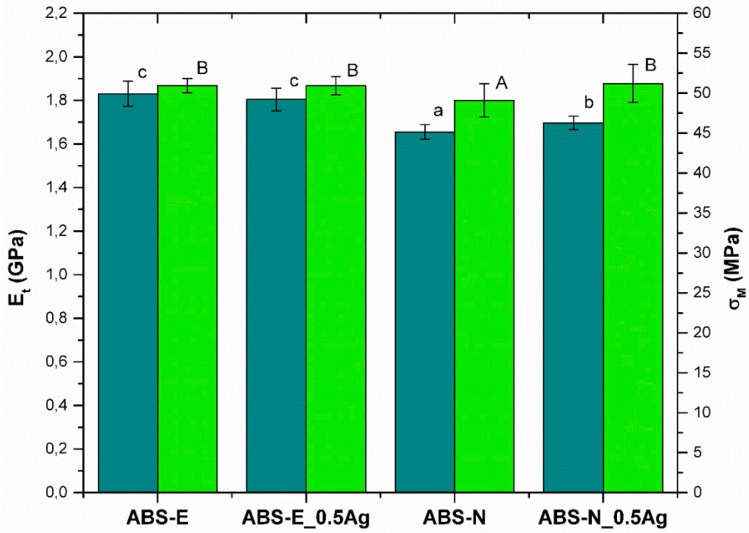
Young’s modulus (E_t_) and tensile strength (σ_M_) of pure polymers and polymers containing silver nanoparticles. Statistically significant differences (*p* < 0.05) between the tested materials are marked a-c for E_t_ and A-B for σ_M_, respectively.

Figure 4 shows the Young’s modulus (E_t_) and the tensile strength of materials (σ_M_). The material based on the Elix M205FC polymer (ABS-E) showed significantly higher values of E_t_ and σ_M_ compared to materials based on the Novodur HD 15 polymer (ABS-N). The presence of silver nanoparticles in the ABS-N material matrix caused a statistically significant increase in strength parameters. On the other hand, in the case of ABS-E_0.5Ag material, no significant changes were observed in comparison to the unmodified material.

### 3.4. Cell Viability/Proliferation Test

**Figure 5:**
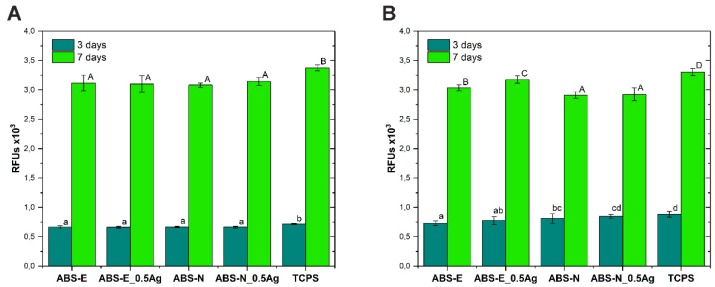
Metabolic activity of Saos-2 (**A**) and Hs680.Tr (**B**) cells after 3 and 7 days of culture in direct contact with the materials tested in the Presto-Blue test. The results are presented as mean values ± standard deviation. Statistically significant differences (*p* < 0.05) between the tested materials are marked a-d for 3-day culture and A-D for 7-day culture.

Figure 5A,B shows metabolic activity of Saos-2 (osteoblast) and Hs-680.Tr (fibroblasts) cells after 3 and 7 days of culture, as evaluated by the PrestoBlue test. 

For all the materials, a significant increase in metabolic activity of osteoblast and fibroblast cells was observed after 7 days of culture. This increase indicates high levels of viability and proliferation of both types of cells. After 7 days of experiment, the osteoblast and fibroblast cells cultured on all of the tested materials showed slightly reduced metabolic activity, as compared to the control material (TCPS). In the case of osteoblasts, metabolic activity of cells was on a similar level in both groups of materials (ABS-E and ABS-N) after both culture periods. While in tests conducted with the use of fibroblasts, cells on ABS-N and ABS-N_0.5Ag after 7-day culture exhibited the lowest metabolic activity.

**Figure 6:**
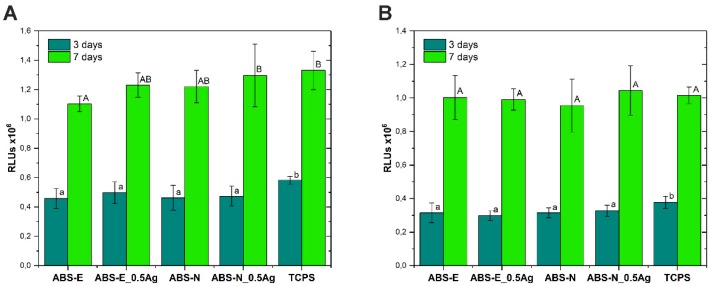
The relative number of Saos-2 (**A**) and Hs680.Tr (**B**) cells after 3 and 7 days of culture in direct contact with the tested materials in the ToxiLight test. The results are presented as mean values ± standard deviation. Statistically significant differences (*p* < 0.05) between the tested materials are marked a-b for 3-day culture and A-B for 7-day culture.

Figure 6A,B shows the relative number of cells of both cell lines after 3 and 7 days of culture in direct contact with the test materials determined in the ToxiLight test.

The results of the study indicate a significant increase in the relative number of both fibroblasts and osteoblasts after 7 days of the experiment. The results again confirm that the materials promote cells proliferation. Similar results were obtained for both polymer materials. For both cells lines, the number of cells grown on all of the materials for 3 days was significantly lower, as compared to TCPS. In turn, after 7-day culture there were no significant differences between the tested samples and control material (besides Saos-2 cells cultured on ABS-E sample). In the case of Saos-2 cells, for material containing silver nanoparticles (ABS-E_0.5Ag and ABS-N_0.5Ag) an increase in the number of cells at 7 days of culture was observed in comparison to pure polymers (ABS-E and ABS-N), however the changes were not statistically significant. Furthermore, the number of osteoblasts grown on both composite materials for 7 days was on similar level, as compared to TCPS. In addition, due to the size of nanomaterials, their additive can modulate cell-material interaction, improving biocompatibility.

### 3.5. Cytotoxicity Test

**Figure 7:**
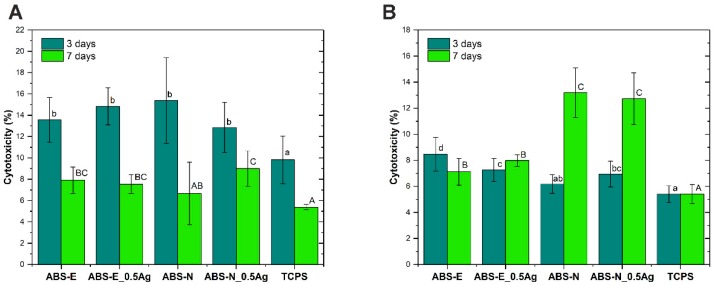
Cytotoxicity of materials evaluated in contact with Saos-2 (**A**) and Hs680.Tr (**B**) cells. The results are presented as mean values ± standard deviation. Statistically significant differences (*p* < 0.05) between the tested materials are marked a-d for 3-day culture and A-C for 7-day culture.

Figure 7A,B shows the cytotoxicity of materials after 3 and 7 days of Saos-2 and Hs-680.Tr cell culture, evaluated on the basis of the level of AK adenylate kinase released into the culture medium during cell culture.

In the case of the Elix polymer for osteoblast cells, the cytotoxicity level for ABS-E and ABS-E_Ag0.5 decreased after seven days of culture. The measured values for both materials were slightly higher for TCPS but did not exceed 16% after three days of experiment and 9% after seven days. In the case of fibroblast lines cultured on the ABS-E group of materials, a slight increase in cytotoxicity was observed with respect to the control material; however, these values did not exceed 9% after both culture periods. In the case of the Novodur polymer for osteoblast cells, the cytotoxicity level for ABS-N and ABS-N_Ag0.5 decreased after seven days of culture. The measured values for both materials were slightly higher than for TCPS and the ABS-E group of materials but did not exceed 16% after three days of experiment and 10% after seven days. In the case of fibroblast lines cultured on the ABS-N group of materials, a slight increase in cytotoxicity was observed with respect to the control material after three days of cell culture. However, these values did not exceed 9%. For the seven-day period, the cytotoxicity increased up to 14%. This behaviour was observed for both pure polymer ABS-N and composite material ABS-N_0.5Ag. The results showed that the materials do not exhibit cytotoxic effects, while encouraging the proliferation of fibroblasts and osteoblasts.

### 3.6. Cell Cultures Observation

**Figure 8:**
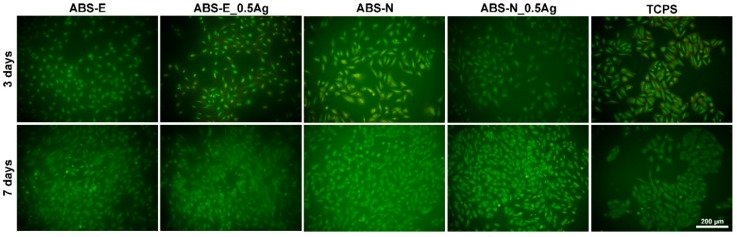
Fluorescence microscope images of Saos-2 cells in direct contact with the tested materials. Magnification 20×.

**Figure 9:**
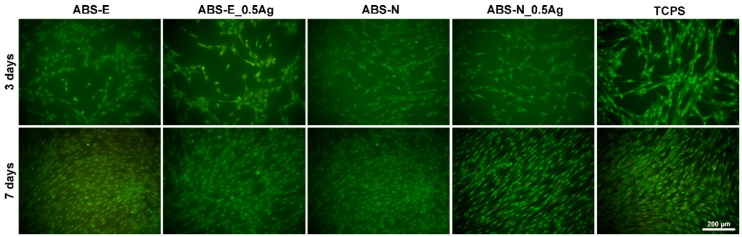
Fluorescence microscope images of Hs680.Tr cells in direct contact with the tested materials. Magnification 20×.

Based on the images obtained from the fluorescence microscope shown in Figure 8 and Figure 9, both osteoblast and fibroblast cells were observed to have proper morphology and were evenly spread homogeneously on ABS-E, ABS-N, and their composites modified with silver nanoparticles surfaces. 

For all materials, a significant increase in the number of Saos-2 and Hs 680.Tr cells was observed after seven days of culture. This increase confirms that the high level of proliferation of both types of cells. The number of cells adhered to both the examined materials, evaluated using microscopic photographs, was similar to that observed for the control material (TCPS). This result is correlated with the dependencies shown in the PrestoBlue and ToxiLight tests.

**Figure 10:**
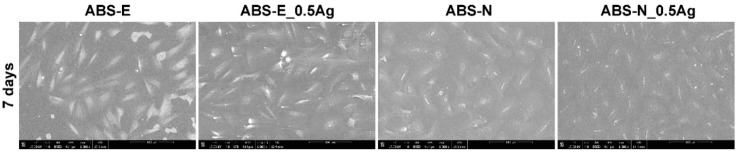
SEM images of Saos-2 cells in direct contact with the tested materials. Magnification 1000×.

**Figure 11:**
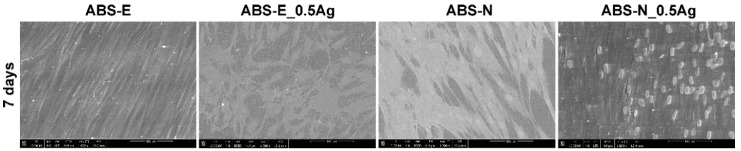
SEM images of Hs680.Tr cells in direct contact with the tested materials. Magnification 1000×.

Based on the images obtained from the scanning electron microscope shown in Figure 10 and Figure 11, both osteoblast and fibroblast cells were present all over the observed regions. 

However, more cells were observed after seven days of cells culture, which correlates with the cell viability presented in Figure 5 and Figure 6 and cell morphology in Figure 8 and Figure 9. Osteoblast and fibroblast cells spread over the material surfaces and showed flattened and proper morphology. The cellular network was formed by well-established, multiple cytoplasmic extensions, providing cell–cell and cell–surface interactions. Silver nanoparticles did not influence the number of cells adhering to the polymers. All the observed regions appeared similar for pure polymers and composites.

## 4. Discussion

According to the work of Ziąbka and co-workers [26,27], the nanomodifier changes the surface properties of the polymer matrix, primarily causing an increase in roughness and surface area. The surface topography has a direct effect on cell adhesion, proliferation, and differentiation, as well as the expressions of the extracellular matrix proteins in direct contact with the material. Materials with high surface roughness have been shown to promote contact osteogenesis [28]. Osteoblast-biomaterial interactions directly affect the development of the bone–implant interface. Zareidoost et al. [29] showed that cells cultured for 3, 7, and 14 days on samples with rough surfaces exhibited a higher cell proliferation rate compared to polished and chemically modified material surfaces. 

Webster et al. [30] noticed that nanostructured surfaces are capable of adsorbing more vitronectin. They observed that surfaces with nanoscale topography were more favorable to osteoblast adhesion compared to the other cell types. Puckett et al. [31] established that the lower the width of the nano rough regions of the patterned substrates (from 80 to 22 μm), the lower the number of adhered osteoblasts. Furthermore, osteoblast adhesion was improved on the deposited nano rough Ti region compared to the non-treated region with microscale topography, regardless of width. This suggests that osteoblasts are able to recognize surface roughness. However, surfaces exhibiting more nano characteristic dimensions favor osteoblast adhesion.

In our work, we did not notice a correlation between roughness and number of adhered cells because the addition of silver nanoparticles did not change the Ra and Rq parameters. Therefore, the cell numbers for pure polymers and composites were comparable. Furthermore, the human cell diameter is 10–15 microns for fibroblasts and 20–30 microns for osteoblasts, which may suggest that nano roughness becomes too insignificant for fibroblasts and osteoblasts to affect cell behavior, such as attachment and proliferation rate. Some research has been conducted on fibroblast behavior in contact with surfaces exhibiting nanotopographical features. Some of these studies revealed the positive impact of surface topography on cell adhesion, whereas another showed the adverse effects of nanostructures [32]. Khang et al. observed that increased cellular function can be related to modified surface wettability resulting from the presence of nanofeatures. In turn, this can affect surface energy, therefore promoting adsorption of proteins, such as fibronectin and vitronectin, containing cell adhesion motif (RGD). Modified surface wettability also stimulates subsequent cellular functions (e.g., extracellular matrix deposition) [33]. Maschhoff et al. showed that the density was the highest for fibroblasts cultured for 18 h on ultrasonicated ZnO/PVC nanocomposites modified with the smallest ZnO nanoparticles [34]. Nanocomposites with smaller nanoparticles (10 nm in diameter) promoted fibroblast proliferation and simultaneously reduced bacterial adhesion and propagation. In our research, we observed wettability changes occurring with the addition of AgNPs, but the nanoparticles we incorporated into the ABS polymers were 80 nm in diameter, which might not affect cell number. There is no precise strength requirements for the materials used for middle ear prostheses. The implant material should have mechanical properties that are as close as possible to those of the host tissues. Since the chain of auditory ossicles is a complex structure with a wide range of Young’s modulus for particular elements (ligaments, muscles, joint, and bones), ranging from 0.049 MPa for ligaments to 14 GPa for bones, it is difficult to design a perfect material for its reconstruction [35]. For this reason, all the materials tested in this study perform biomechanical functions and may be used for middle ear implants.

## 5. Conclusions

In conclusion, the cytotoxicity, viability, and proliferation tests conducted using fibroblast and osteoblastic cells showed that the tested poly(styrene-acrylonitrile-butadiene) polymers from ELIX Polymers and INEOS Styrolution are highly biocompatible. Furthermore, the results suggest that these materials possess sufficient mechanical properties for ossicular chain reconstruction. The addition of 0.5% by weight of silver nanoparticles did not significantly affect their biological and mechanical properties. The surface roughness of composite materials was not significantly affected, while wettability was slightly improved. Relative numbers of osteoblast and fibroblast cells did not change upon polymer modification with a nanomodifier. This study demonstrates that both pure polymers and composites are biocompatible, suggesting that the nanocomposites may successfully be used as a medical implant material, such as for middle ear prosthesis.
